# Factors Affecting Toxic and Essential Trace Element Concentrations in Cow’s Milk Produced in the State of Pernambuco, Brazil

**DOI:** 10.3390/ani13152465

**Published:** 2023-07-30

**Authors:** Emanuel Felipe de Oliveira Filho, Marta López-Alonso, Guilherme Vieira Marcolino, Pierre Castro Soares, Carlos Herrero-Latorre, Carla Lopes de Mendonça, Nivaldo de Azevedo Costa, Marta Miranda

**Affiliations:** 1 Department of Veterinary Medicine, Universidade Federal Rural de Pernambuco (UFRPE), Rua Dom Manoel de Medeiros, s/n, Dois Irmãos, Recife 52171-900, Brazil; felipe130188@gmail.com (E.F.d.O.F.); pcastro.pe@gmail.com (P.C.S.); 2 Department of Animal Pathology, Faculty of Veterinary, Campus Terra, University of Santiago de Compostela, 27002 Lugo, Spain; marta.lopez.alonso@usc.es; 3 Health and Reproduction of Ruminants-UAG/UFRPE, Garanhuns 55292-270, Brazil; marcolinogv@outlook.com.br; 4 Research Institute on Chemical and Biological Analysis, Analytical Chemistry, Nutrition and Bromatology Department, Faculty of Sciences, Campus Terra, University of Santiago de Compostela, 27002 Lugo, Spain; carlos.herrero@usc.es; 5 Clinic of Cattle of Garanhuns/UFRPE, Campus Garanhuns, Av. Bom Pastor–Boa Vista, Garanhuns 55292-270, Brazil; carlalopes.mendonca@gmail.com (C.L.d.M.); na.costa@hotmail.com (N.d.A.C.); 6 Department of Anatomy, Animal Production and Clinical Veterinary Sciences, Faculty of Veterinary, University of Santiago de Compostela, 27002 Lugo, Spain

**Keywords:** milk, cattle, toxic and essential trace elements, Pernambuco, Brazil

## Abstract

**Simple Summary:**

Milk is one of the main components of the human diet, mainly due to its mineral and protein content. But its contamination by heavy metals could produce a serious public health problem. In this study, we have determined the levels of toxic (Cd and Pb) and essential (Cu, Fe, and Zn) elements in raw milk from cows raised in the State of Pernambuco (Brazil). A high percentage of the samples had Pb levels above the tolerance limit established by Brazilian legislation, and the proximity of the farms to major roads is the main cause. Therefore, the consumption of milk produced under these conditions can be considered a risk to public health.

**Abstract:**

The aim of this study was to provide information on the levels of toxic (Cd and Pb) and essential (Cu, Fe, and Zn) elements in cow’s milk produced in the State of Pernambuco (Brazil). A total of 142 samples of raw milk were collected, and the concentrations of essential and toxic elements were determined using inductively coupled plasma-optical emission spectrometry. In almost 30% of the samples analyzed, the Pb content exceeded the maximum level established in the Brazilian legislation (0.05 mg/L). By contrast, in all the samples, the Cd content was below the maximum allowable level (0.02 mg/L). The essential trace elements Cu, Fe, and Zn were generally present at lower concentrations than reported in other studies and can be considered within the deficient range for cow’s milk. Statistical and chemometric procedures were used to evaluate the main factors influencing the metal concentrations (proximity to major roads, presence of effluents, and milking method). The study findings demonstrate that the proximity of the farms to major roads influences the concentrations of Cd, Pb, and Cu and that this is the main factor explaining the Pb content of milk. In addition, the presence of effluents influenced the concentrations of Cu, while no relationship between the metal content and the milking method was observed. Thus, in accordance with the study findings, the consumption of cow’s milk produced in the region can be considered a risk to public health due to the high concentrations of Pb and the low concentrations of other essential minerals such as Cu, Zn, and Fe in some of the milk samples.

## 1. Introduction

Milk is a wholesome food and represents an important constituent of the human diet (especially for infants, schoolchildren, and the elderly) as it contains nutrients that are essential for growth, bone development, immune function, and other important physiological functions [1,2]. Milk is considered the most diverse natural food product in composition. In addition to being a good source of protein, fat, and carbohydrate, milk is an ideal source of macro- and microelements such as calcium (Ca), potassium (K), phosphorous (P), magnesium (Mg), zinc (Zn), iron (Fe), copper (Cu), manganese (Mn), and selenium (Se). However, milk can also contain toxic elements, the most important of which is lead (Pb), which is known to have deleterious effects on the developing nervous system of children [2,3]. Milk can also be contaminated by other toxic metals such as cadmium (Cd), mercury (Hg), arsenic (As), and nickel (Ni), and even by high concentrations of essential elements (such as Co, Cr, Cu, Fe, and Zn) [4,5].

The composition of milk is greatly influenced by the nutritional status of the cow, as well as the stage of lactation, management, productive stage, genetics, and breed [6]. Moreover, the essential trace element profile of milk, particularly toxic element residues, is largely affected by the environment where the cows are raised [3,5,7]. Heavy metals mainly enter cow’s milk through cattle feed and drinking water (as well as via the atmosphere). The feed and water can, in turn, be contaminated through the soil via sewage sludge used as fertilizer, artificial fertilizers, metals used in fungicidal agents, and other agricultural chemicals, and also via wastewater from various industries. The risk of milk becoming contaminated is particularly high in areas affected by anthropogenic pollution, such as smelting or mining areas and highly industrialized regions, allowing for the transfer of metal contamination to the atmosphere, soil, water, animal feed, animals and their products, and finally to humans [2,3,5,8,9,10]. In the case of Pb, milk can become contaminated when cows graze and drink water at roadsides. In addition, factors related to the manufacturing practices (particularly hygiene during milking) and possible contamination from the equipment during processing [4,5] can also increase the concentration of this toxic element in milk. In recent years, the contamination of milk by toxic elements (particularly Pb) has been indicated to be one of the most serious aspects of environmental pollution for human health, because milk is widely consumed, especially by children [3]. Numerous studies have therefore been conducted in order to monitor the presence of toxic elements in milk and related products (see table of previous studies), particularly in developing countries where information from national monitoring programmes is scarce and legislation aimed at environmental protection is sometimes less restrictive than in other more developed countries.

The concentrations of metals in milk sometimes exceed the maximum limits recommended. According to the European Commission [11] and Codex Alimentarius Commission, the maximum limit for Pb is 0.020 mg/L, but the Brazilian legislation (Decree nº. 55871/65) [12] establishes a higher limit of 0.05 mg/L. Previous studies carried out in different states of Brazil indicated that Pb concentrations in milk exceeded the maximum limits [13,14,15]. However, at present there are no data on the concentrations of toxic and trace elements in cow’s milk produced in the semi-arid region of Pernambuco. The study of the transfer of Pb to the rock–soil–plant–milk systems is essential in view of the high natural levels of Pb in the rock and soil in this region [16,17,18]. The dairy basin in the state of Pernambuco plays an important role in the local economy and in supplying milk, since it is an important producing area where milk is treated industrially and is also sold at fairs and local markets [17]. The rearing system in most of the properties in the region is extensive or semi-intensive, and some of the Pb ingested by cows via the consumption of contaminated forage could be transferred to the milk [16,17,18].

The main objective of the present study was to provide information about the levels of toxic (Cd and Pb) and essential (Cu, Fe, and Zn) elements in cow’s milk produced in the State of Pernambuco and to determine the main factors influencing the concentrations of these elements (e.g., proximity to road, presence of effluents, and the milking method) to evaluate whether the consumption of cow’s milk produced in the region can be considered a public health risk.

## 2. Materials and Methods

This study was conducted according to the guidelines of the Declaration of Helsinki and approved by the Ethics Committee of The Universidade Federal Rural de Pernambuco (protocol code 23082.009185/2017-15, approved 15 April 2017).

### 2.1. Sample Collection

**Figure 1:**
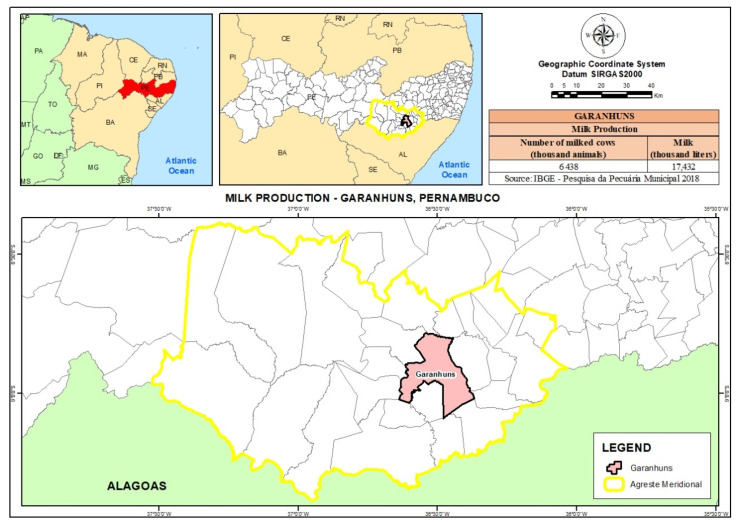
Municipality of Garanhuns and milk production in the Pernambuco dairy basin.

The milk samples analyzed in this study (*n* = 142) were produced by cows raised in extensive and semi-intensive dairy farm systems in the mesoregion of Agreste de Pernambuco (Garanhuns microregion) (Figure 1). A total of 14 farms were visited and the samples were collected between September and December 2018. The animals were raised in corrals or paddocks, divided according to the organization of the owner, and were fed with native pasture, forage palm, corn, and soybean, with water and mineralized salt ad libitum. The distance from the farm in which each sample was taken and the main roads were calculated. The proximity of the farms to major roads was considered close when the distance was less than 3 km and distant otherwise. The farms were in a radius of 1 to 12 km to the main roads. Five farms (40 samples) were close, and the remaining nine (102 samples) far. Regarding effluents, the influence of hydrographic features (large and small rivers, as well as springs and other sources such as wells and dams) on the farms and access to these sources by the cows was taken into account. Eight farms (66 samples) had influence of effluents and the remaining six (76 samples) did not. The milking method was classified as manual (79 samples), performed manually by a responsible worker, or automatic (63 samples), carried out by means of milking machinery. Raw milk samples (15 mL each) were obtained from cows in the early lactation stage (11 to 100 days postpartum). The first 3 jets of milk were discarded and the next 15 mL was collected. Samples were stored at 4 °C in 15 mL sterile plastic containers with a lid and sent to the Clinical Pathology Laboratory of the Garanhuns Cattle Clinic (Universidade Federal Rural de Pernambuco, UFRPE), where they were then stored at −20 °C until analysis.

### 2.2. Reagents and Standard Solutions

All solutions were prepared using ultrapure water of resistance 18 MΩ cm^−1^ produced by a Milli-Q purification system (Millipore Corp., Bedford, MA, USA). Stock standard solutions of the elements (1000 mg/L) were of ultrapure grade (ICP Multi element standard solution IV certiPUR). Nitric acid (69%) was obtained from Merck (Poole, UK).

### 2.3. Sample Analysis

Samples were subjected to acid digestion before analysis. The microwave-assisted digestion procedure was carried out at the Research Support Center (CENAPESQ) of the Federal Rural University of Pernambuco (UFRPE). Briefly, each sample of raw bovine milk (5 mL) was placed in a glass flask with 10 mL of HNO3 and digested in a microwave oven (model MarsXpress-CEM Technology Inside) for 28 min (step 1: 110 °C for 8 min, step 2: 170 °C during 10 min, and step 3: 170 °C for 10 min.) Digested samples were filtered through pyramid-folded filter paper (weight 80 g m^2^, filtration rate 20–25 s) into a new sterile tube.

The concentrations of Cd, Pb, Cu, Fe, and Zn were determined using atomic emission spectrometry with inductively coupled plasma (ICP-OES) (Optima 7000 DV, PerkinElmer, Waltham, MA, USA) in the Soil Chemistry Laboratory (DEPA) (UFRPE). All samples were analyzed in duplicate, and the concentrations were expressed in mg/L.

An analytical quality control programme was applied throughout the study. Blank samples were run alongside the test samples and the values thus obtained were subtracted from the sample readings. The limits of detection (LOD) in the acid digest were calculated as three times the standard deviation of the reagent blanks: 0.012 (Cd), 0.036 (Pb), 0.14 (Cu), 0.85 (Fe), and 0.93 (Zn) µg/L. The elemental concentrations of all samples analyzed were above the respective LODs. To check the accuracy of the analytical method, multi-element standard solutions were used for calibration and run with the samples. The precision of the method was expressed as the analytical recovery, which in all cases was within an acceptable range (90 to 110%), with a relative standard deviation (RSD) <10%.

### 2.4. Statistical and Chemometric Analysis

An *X*_142×5_ matrix was used to analyze the data, with the rows corresponding to the 142 milk samples and the columns to the contents of the 5 toxic and essential metals determined using ICP-OES. Other information regarding the factors evaluated (proximity to main roads, presence of effluents, and milking system) were also included as qualitative variables in the data matrix. The data distribution was checked using the Kolmogorov–Smirnov test; as the data were not normally distributed, they were log-transformed before analysis and presented as geometric means. A general linear model (GLM) was used to evaluate the effect of the proximity of the farm to main roads (0: no; 1: yes); the presence of effluents (0: no; 1: yes); milking method (0: automatic, 1: manual); and their interactions in the toxic and essential trace element concentrations in milk. The statistical analyses were performed using IBM SPSS for Windows v.24 (IBM Corporation, Armonk, NY, USA) and test results were considered statistically significant at *p* < 0.05.

In addition, two unsupervised chemometric techniques, principal component analysis (PCA) and hierarchical cluster analysis (HCA), were used to reveal the latent structures residing in the data set and to evaluate the relationship between samples and variables. PCA was used to display the information contained in the data in a reduced dimension with minimum loss of data variance, and HCA (an unsupervised display chemometric technique, often used to complement PCA) was used to establish clusters of samples (and variables) based on the distance measures between them in the 5-multidimensional space [19]. All chemometric techniques were carried out using Statgraphics Centurion XVI v.16.1.15 (Statistical Graphics Corporation, Rockville, MD, USA).

## 3. Results and Discussion

### 3.1. Toxic and Essential Trace Element Concentrations in Milk

The toxic and essential trace element concentrations determined in the milk samples are presented in Table 1. The concentrations of these elements determined in other studies around the world are shown in Table 2 for comparative purposes.

The mean Cd and Pb concentrations determined in the present study were 0.007 and 0.043 mg/L, respectively. The mean Cd concentrations were low and similar to those reported in other studies in unpolluted areas (generally below 0.010 mg/L) and much lower than those reported in previous studies in Brazil [14,20] and in polluted areas of developing countries in Asia, Africa, and South America (see Table 2). However, the concentrations of Pb are higher than those reported in other recent studies conducted in relatively unpolluted areas from Europe or North America (see Table 2), but lower than those reported in previous studies in Brazil [13,14,15,20]. The Pb concentrations determined in the present study are similar to those determined in some polluted regions of Iran [8] and Peru [21], but much lower than those reported in other polluted areas in Asia or Africa (see Table 2).

Considering the applicable legislation, the Pb content of 29.6% in the samples (42/142) was above the limit permitted under Brazilian law (Decree n. 55871/65), which is 0.05 mg/L [12], and 97% of the samples (138/142) exceeded the limit established in the European Union [11] and Codex Alimentarius Commission and the WHO, i.e., 0.020 mg/L. By contrast, the Cd content of all the samples was lower than 0.02 mg/L, the limit established by Brazilian law [12].

Previous studies conducted in Brasilia [22], Paraná [14], and Sao Luis [15] also reported Pb concentrations higher than the maximum limit. The Pb and Cd contents of milk depend on the proximity of polluted areas, crowded roads, the level of industrialization (see Table 2), and are also influenced by the control and legislation limits [3]. The concentrations determined in Western and Central Europe, the USA, and Canada are below levels considered to represent a risk, unlike in Brazil, Mexico, Peru, some parts of Asia or Africa, and polluted areas of Eastern Europe [3,23]. Recent studies have reported Pb and Cd concentrations in milk higher than 60 and 12 mg/kg, respectively, in some parts of India [3]; Pb concentrations in milk exceeding 13 mg/kg have been reported in Indonesia [24].

Considering the trace elements, the mean concentrations of Cu (0.020 mg/L), Fe (0.055 mg/L), and Zn (0.621 mg/L) were generally lower than those reported in other studies (see Table 2) and, in the case of Cu and Fe, can be considered within the deficient range for cow milk according to Puls [25] (deficient ranges are Cu: 0.010–0.020; Fe: <0.2; and Zn: <0.5 mg/L; adequate ranges are Cu: 0.05–0.6; Fe: 0.2–0.63; and Zn: 2.3–4 mg/L). These results indicate that milk produced in this region is not a good source of trace elements for the local population. The mean levels of Cu and Fe in raw cow’s milk samples across the world ranged from 0.0136 to 36 mg/L and from 0.33 to 16.4 mg/L, respectively [3]. The presence of heavy metals such as Pb and Cd is associated with changes in the trace mineral profile of milk and negatively affects the nutritional quality of the product, e.g., by reducing the Fe content [2]. Trace element deficiencies in livestock are prevalent in different regions of Brazil [26], especially in several semi-arid climate areas. During the dry season, the pastures are generally overgrazed, leading to mineral deficiencies in the herds [16].

**Table 2:** Toxic and essential trace element concentrations (in mg/L) in cow’s milk as determined in previous studies. Values are arithmetic means expressed in mg/kg or mg/L wet weight.

Country	Region	Cd	Pb	Cu	Fe	Zn	Reference
Algeria	Guelma area; polluted area	0.03	0.94	0.14	0.76	4.02	[27]
Algeria	Polluted area	0.030		0.239	1.43	5.98	[28]
Argentina	Rural areas Southeast of Córdoba		0.0023	0.0380	0.855	1.800	[29]
Bangladesh	Dairy Farms	0.024	0.015	0.064	0.333		[30]
	Small household	0.047	0.012	0.127	0.631		
Brazil	Paraná state; Pasteurized	0.018	0.281				[14]
	Paraná state; In Natura	0.031	0.181				
Brazil	State of Goiás (supermarkets)	0.05	0.24	0.49	0.96	3.73	[20]
Brazil	Industrial area	0.002	ND	0.063		3.87	[31]
	Non-industrial area	0.003	0.003	0.068		3.15	
Brazil	Vale of Paraíba region		0.23	1.73	1.05	4.59	[13]
China	Industrial	0.00015	0.00286				[32]
	Unpolluted	0.00013	0.00232				
China	Samples from Shandong and Shaanxi cities	0.00007	0.0014	0.0324	0.352	3.234	[33]
China	Ten main milk producing areas in China	0.00005	0.00175				[34]
Croatia	Four unpolluted areas	ND	ND–0.0071	0.06–0.07	0.26–0.30	3.7–4.8	[35]
Egypt	Beni Suef governorate	0.051	0.214	0.0953	8.994	6.29	[36]
England	Southern England; conventional farmland			0.0606	2.03	5.00	[37]
	Southern England; organic farmland			0.0524	0.66	4.51	
Ethiopia	Kosoye Amba-Rass, Tana-Abo, and Nara-Awdarda, North Gondar, Amhara Regional State	0.29	0.15	1.12		3.02	[38]
Hungary	Highway area	0.005	0.025	0.336	0.797	1.494	[39]
	Non-polluted	ND	0.012	0.137	0.788	2.241	
India	Mining areas		0.09–0.13	0.31–0.51	8.8–11.4	1.22–1.04	[40]
India	Ladakh, a trans-Himalayan high-altitude region	0.007–0.009	0.005–0.006	0.23–0.30	3.55–4.91	1.99–3.76	[41]
India	Industrial areas	0.02–0.07	0.05–0.20	0.07–0.35		1.22–20.94	[42]
India	Industrial area	0.096	0.480	0.090	3.97	6.09	[2]
	Non-industrial area	0.033	0.250	0.101	5.10	3.95	
Indonesia	City area, Padang	ND	13.6–20.6	1.17–2.17		28.8–53.1	[24]
Iran	Farms close to petroleum industries	0.0047	0.047				[8]
Iran	Arak city	0.00395	0.0125				[43]
Iran	Industrial regions of Iran	0.00111	0.0140	0.427		0.571	[44]
Iran	Lorestan province	0.10	2.72	0.14		3.07	[45]
Italy	Industrial area	ND	0.02	0.07–0.08	14.5–16.8	2.21–2.86	[46]
Italy	Calabria	0.0002	0.001	0.003		2.02	[1]
Kazakhstan	Almaty region; unpolluted	0.0027	0.0045				[47]
Korea	Supermarkets	0.00238	0.00335	0.3834		4.754	[48]
Kosovo	Rural areas	0.001	0.0017	0.018	0.426	3.151	[49]
Mexico	Areas irrigated with wastewater		0.03	0.01		0.71	[9]
Mexico	Puebla, industrial wastewater	0.002	0.024	0.030			[50]
Moscow	Moscow region	0.004–0.011	0.075–0.110	0.11–0.21	0.55–0.82	1.21–141	[51]
Pakistan	Sargodha; near traffic road	0.04–0.3	0.3–0.8				[52]
Peru	Near metallurgical complex	0.020	0.058				[21]
Peru	Mining-metallurgical industries	0.018	0.577				[10]
Poland	Lubuskie ProvinceOrganic farms	0.003–0.004	0.037–0.041	0.038–0.045	0.198–0.258	3.02–3.28	[53]
Poland	Low-level industrialization	<0.004	0.012	0.360		4.83	[54]
	High-level industrialization	<0.004	0.234	1.33		15.84	
Romania	Intermediate-level industrial	0.0039	0.120	2.4		4.8	[55]
	Intensive industrial	0.007	0.577	0.837		4.8	
	Small cattle farms	0.007	0.024	0.265		3.18	
Romania	No industry	0.006	0.066	0.30		2.5	[56]
Serbia	Novi Sad (Vojvodina) market	0.00349	0.0754	0.118			[57]
Spain	Organic farms	0.000135	0.000653	0.041	0.425	3.326	[58]
	Conventional farms	0.000098	0.000516	0.051	0.395	3.639	
	Conventional (supermarket)	0.000087	0.000267	0.069	0.351	3.933	
Spain	Unpolluted region; organic	ND	0.000519	0.039	0.271	2.851	[7]
	Unpolluted region; conventional	ND	0.000389	0.048	0.301	3.368	
Spain	Farms near mining and industrial area and highway traffic	<0.002	0.004				[59]
Sri Lanka	Four agro-climatic zones	0.001–0.002	0.005–0.02	0.02–0.12	0.49–3.15	1.49–2.93	[60]
Turkey	Local markets in the city of Edirne			0.138	3.1	3.4	[61]
Turkey	Iğdır City	0.0001–0.004	0.050	0.08–1.80		2.21–32.5	[62]
Turkey	Close to highways	0.39	1.85	0.62	4.2	1.85	[63]
Zambia	Farms near mining area		0.002				[64]

ND: not detected.

### 3.2. Effects of Proximity to Main Roads, Presence of Effluents, and Milking Method on Toxic and Trace Element Concentrations in Milk

A general linear model was applied to the data in order to evaluate the effect of factors that potentially influence the toxic and trace element concentrations in milk samples (proximity of the farm to major roads, presence of effluents in the vicinity of farm, and the milking method). The results are presented in Table 3, and it can be seen that the proximity to major roads (R) had a significant influence on the concentrations of Cd, Pb, and Cu in the milk samples. The presence of effluents in the vicinity of farms (E) only influenced the Cu concentration, while the milking method (M) did not influence the metallic profile of the samples.

**Figure 2:**
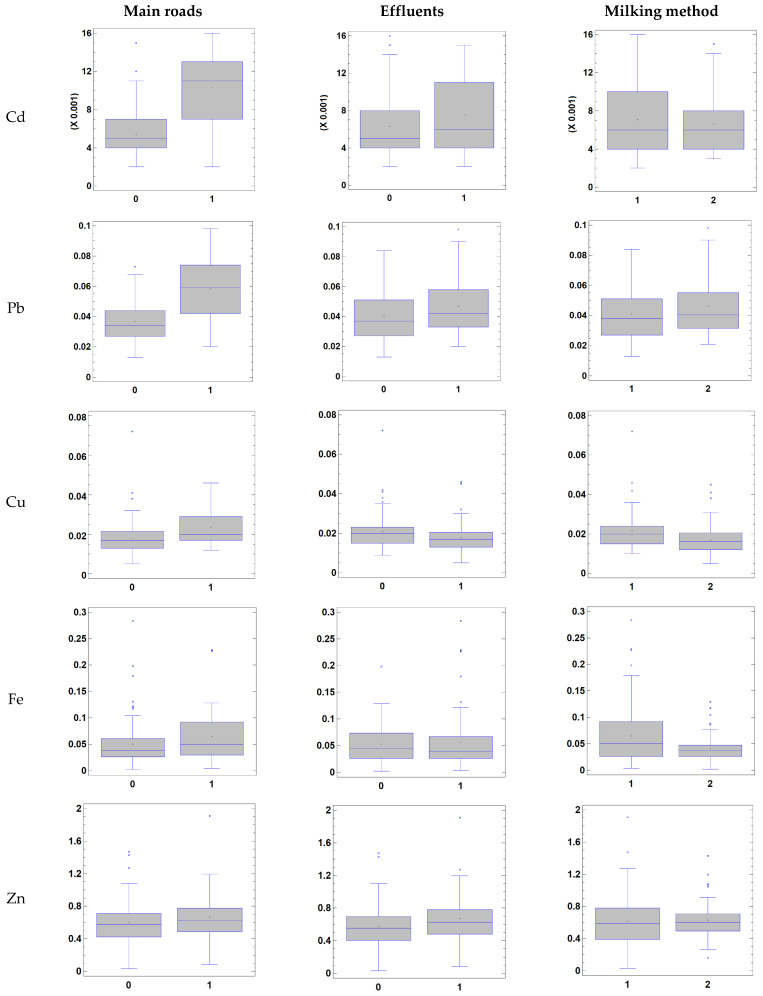
Box and whiskers plot showing the effect of proximity of farms to main roads, the presence of effluents, and milking method on toxic and trace element concentrations in milk (in mg/L).

The proximity of the farm to major roads was the most important factor in the analysis, exerting a significant effect on the Cd, Pb, and Cu concentrations in milk, which were 94, 90, and 32% higher in the milk samples from farms close to major roads than in milk samples from farms distant from major roads (Figure 2). The influence of the proximity to major roads on toxic element accumulation in milk, blood, water, soils, forage, and other food products is well known [39,52,65]. Kodrik et al. [39] reported significantly higher levels of Cd (0.005 vs. ND mg/L), Pb (0.025 vs. 0.012 mg/L), and Cu (0.336 vs. 0.137 mg/L) in cow’s milk originating from traffic-intensive areas in Hungary, whereas Fe concentrations (0.797 vs. 0.788 mg/L) were similar to those in milk produced in unpolluted green areas and the Zn concentrations were lower in the former than in the latter (1.494 vs. 2.241 mg/L). Tahir et al. [52] reported high levels of Cd (0.04–0.3 mg/L) and Pb (0.3–0.8 mg/L) in cow’s milk in Pakistan, which were attributed to Cd- and Pb-contaminated feed, air pollution, and drinking water contaminated by dust from areas close to roads. Similar results were reported by Bigucu et al. [63] in areas of Turkey close to major roads. The levels of contamination in the milk were also higher in these studies than in the present study.

Leaded petrol has caused more exposure to Pb than any other source worldwide, contaminating air, dust, soil, drinking water, and food crops, and it has caused harmfully high human blood Pb levels around the world, especially in children [66]. Lead persists in the environment and can bioaccumulate in bodies, with long-term effects lasting after the initial source of Pb has disappeared [66]. Some decades after Pb was banned in petrol, residues of this metal in milk have largely decreased, but emissions of toxic metals associated with motor vehicles are still considered among the most important sources of heavy metals in the environment, and high concentrations of heavy metals are found in soils near roads/highways [67]. Monitoring heavy metal concentrations in roadside environments therefore remains of great importance. The region studied, despite being a relatively unpolluted area without big industries, smelting, or mining areas, has high natural levels of Pb in the rock and soil [16], which could also contribute to the high levels of Pb in cow’s milk in this region.

The presence of effluents was also found to be a significant factor regarding the Cu of the milk, although the samples from farms affected by effluents had lower Cu concentrations (23%) than samples from farms not affected by effluents. No significant interactions between the presence of effluents and the proximity to roads were found, and milk samples from farms close to and distant from main roads had lower Cu contents when they were close to sources of effluents. Concentrations of Cu in milk are known to be influenced by the environment, industrialization, and anthropogenic activities. A large part of the Pernambuco hydrographic basin, specifically the Garanhuns microregion, receives industrial effluents, and the water cannot be used to supply the city, as it contains toxic elements, and the rivers in these areas are contaminated and affected by anthropogenic activities [68]. As in the present study, Ogundiran et al. [69] reported that the Cu content was significantly higher in the milk from cows reared in an industrialized area than in milk from cows reared in unpolluted areas. These findings suggest that other factors/trace elements present in the effluents may interact negatively with the Cu metabolism in cows, leading to a significant reduction in the Cu concentration in the milk. For example, complex non-competitive negative interactions between Cu, sulphur, and molybdenum and competitive interactions with Cd and Zn for the metallothionein binding sites are known to occur in ruminants [6]. Other interactions between Cu and Cd [70,71] and Cu and Pb [72] have also been described in cattle. Increased levels of Cd and Pb interfere with the metabolism of the essential trace elements, in particular with the metabolism of Cu, with calves at risk of Cu deficiency [71]. Such interactions are sometimes difficult to interpret as they depend on the chemical form and relative concentrations of the elements in the environment and can involve more than two elements [2,72].

### 3.3. Chemometric Analysis: PCA and HCA

Chemometric analysis can be used for the detailed examination of large sets of data, enabling the visualization of complex interactions between samples and variables, and among samples and variables. Latent structures and relationships residing in the data matrix are commonly studied by means of two display chemometric techniques: PCA and HCA.

PCA was used for the primary evaluation of the 5-dimension data set. PCA transforms the autoscaled data matrix *X*_142×5_ into a product of two matrices: the score matrix S145xPC, which includes information about the samples, and the loadings matrix LPCxPC, which includes information related to the variables. When the number of principal components (PC) selected is smaller than the number of original variables, PCA produces a visualization of the data matrix in a reduced dimension, simplifies the original problem, and enables an examination of the relationships between samples and between variables through score and loadings plots, respectively [19].

In the present study, PCA was applied to the original autoscaled data matrix, and the first two PCs were considered sufficient to represent all the data. The first two principal components explained 75.38% of the total variance; the remaining PCs yielded eigenvalues <1, indicating poor information content (Table 4).

**Figure 3:**
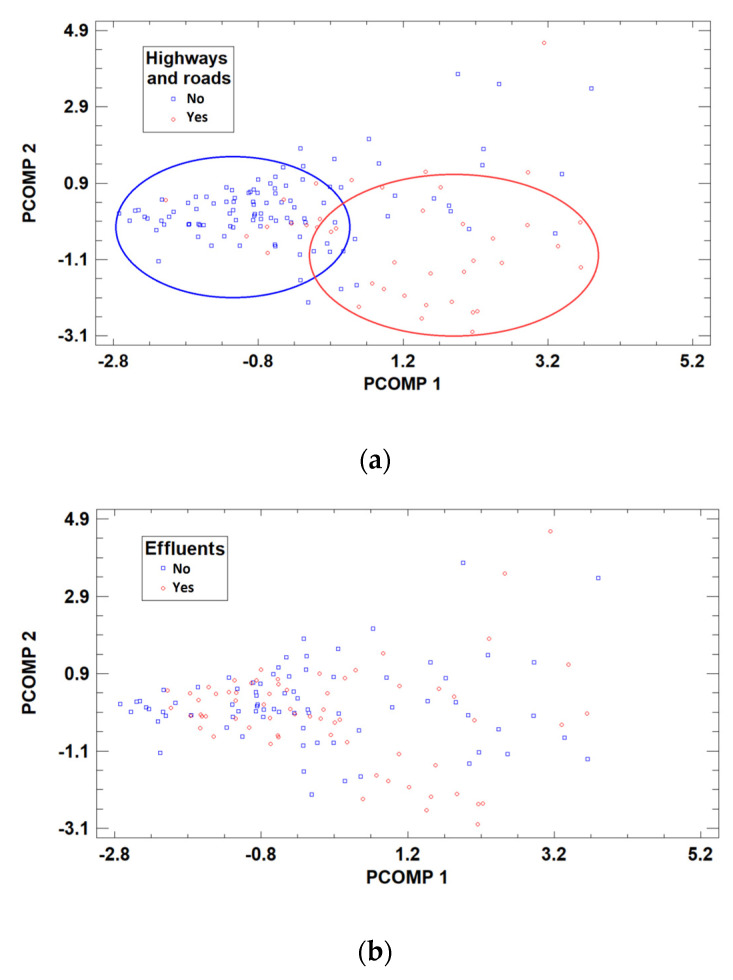

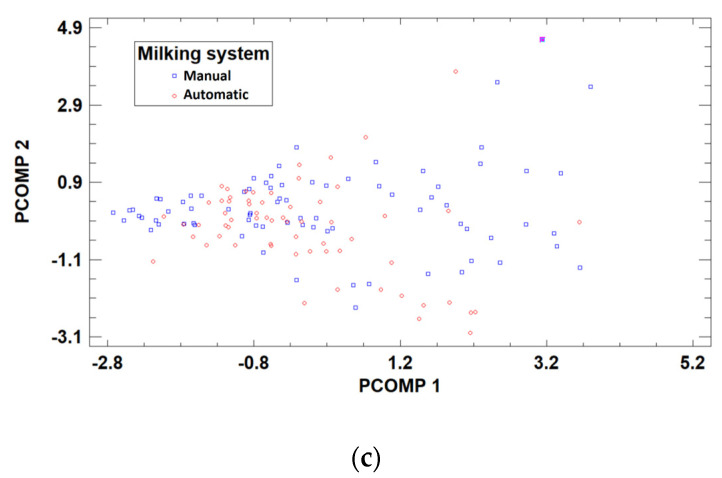
Twodimensional-score plot of the samples obtained using PCA according to (**a**) the proximity the farms to major roads, (**b**) the presence of effluents, and (**c**) the milking method.

This enabled the evaluation of the whole data set by using a 2D-score (or loading) plot, where the samples (or variables) are represented in the space defined by the first two principal components. An examination of the sample scores in this plot produced some interesting results. A natural separation of the samples into two groups according to the proximity of the farms to major roads was detected (Figure 3a), implying an evident influence of the traffic emissions on the metal content of the milk. Despite this “natural” separation, there was also some degree of overlap between the two categories in the 5-multidimensional space of the variables, as seen in the score-plot.

The same separation in groups of samples was not detected for the other two factors studied, i.e., the presence of effluents in the vicinity of the farm (Figure 3b) and the milking method (Figure 3c). These results are consistent with previous findings established by using a GLM to evaluate the effect of the three factors considered, and they confirm that the proximity to major roads was the most significant factor affecting the concentration of metals in milk samples. The fact that the presence/absence of effluents in the proximity of the farms did not distinguish groups, which appeared randomly mixed thereby showing no significant influence of this factor on the metal content studied, reaffirms the possibility that other elements not determined in the study may interact negatively with Cu.

**Figure 4:**
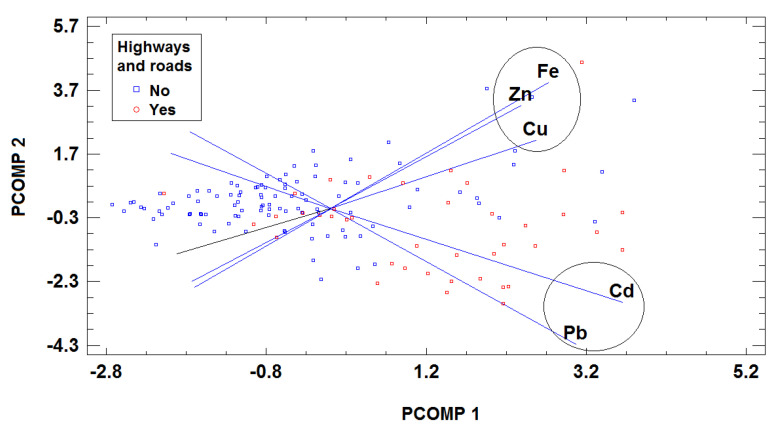
Biplot of the whole data set obtained using PCA. Samples are coded according to the proximity of major roads.

A PCA-biplot was constructed in order to study the relationship between variables in the whole data set. Both the samples and variables of the multivariate data were presented together (as scores and loadings) in the biplots. This type of plot reveals the relationships between the variables and also with the samples or groups of samples. In the case at hand, the 2D-biplot obtained, presented in Figure 4, showed the following: (i) a high degree of correlation between the three essential trace elements (Cu, Fe, and Zn), indicating a possible common origin, and (ii) an important association between toxic trace elements (Cd and Pb), also with a possible shared origin. The source in this case is probably from the emissions of motor vehicles from major roads, because the axis corresponding to these two variables coincided with the direction of the multidimensional space including the milk samples from farms close to major roads.

**Figure 5:**
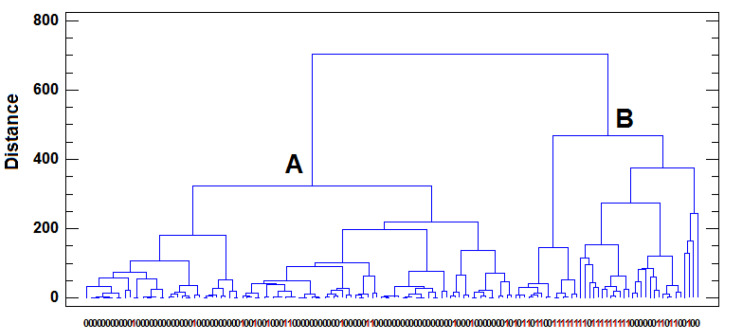
Dendrogram of the samples according to the proximity of major roads obtained using HCA (Squared Euclidean distance and Ward agglomerative method). (0 = far, 1 = close).

The second step of chemometric analysis consisted of using HCA. This unsupervised chemometric procedure searches for groups of samples (or variables) in the multidimensional space. It is based in an algorithm that arranges similar samples (or variables) into groups called clusters. The similarity between objects is calculated on the basis of the distance that separates them, considering that near samples in the 5-dimensional space of the variables will be very similar to each other. In the present case, the similarity was measured as the squared Euclidean distance, and the Ward method was used as an agglomerative algorithm procedure to identify clusters [73]. The dendrogram of the milk samples obtained when HCA was applied to the complete data set in the autoscaled *X*_142×5_ matrix can be seen in Figure 5. An examination of the dendrogram revealed the presence of two clusters of milk samples in the 5-dimensional space defined by the metal variables. The first cluster (A) is composed of samples from farms distant from major roads, while the second (B) mainly includes samples from farms close to major roads. The presence of samples near roads in cluster A and the appearance of samples far from roads in cluster B demonstrate some degree of overlap between clusters previously indicated in the PCA. This separation in two clear groups does not occur for either the milking type or the effluent factors, thus confirming the conclusion reached in the PCA that the most significant factor affecting the metal content of the samples is the traffic in the proximity of livestock farms. Moreover, the group of milk samples from farms far from major roads were more similar to each other than the group of milk samples from farms close to major roads were (Figure 5). This was an expected result as the distance of the farms from major roads was variable. It has been reported that the levels of heavy metal contamination (Pb and Cd are the most widely studied) in both soils and forage decreases to background levels with increasing distance on both sides of major roads [52,66]. Cattle and other animals that are grazed close to roads have been shown to have higher blood Pb levels than animals housed indoors [2,74], and the significant correlation between blood and milk Pb levels increases the potential of human exposure [2,74]. One of the most important causes of high Pb content in milk from rural, relatively unpolluted areas may be the proximity to major roads or transhumance along roads and/or motorways [63,75].

**Figure 6:**
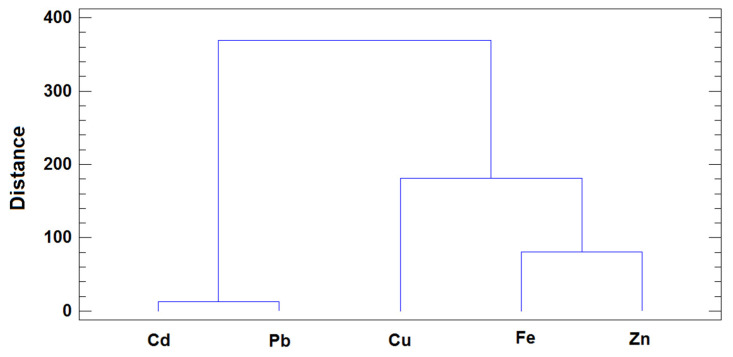
Dendrogram of the variables obtained by HCA (Squared Euclidean distance and Ward agglomerative method).

Additionally, HCA was used to evaluate associations between variables, and in this case, two clusters of variables were also detected (Figure 6): the first included the toxic metals, Cd and Pb, and the second comprised the essential trace elements. The variable arrangement may be directly linked to the differences in these metals according to the presence or absence of any major roads in the proximity of the farm where the milk samples were collected. In the plots, the metals are ordered from left to right on the basis of their capacity to distinguish between samples near and far from main roads. The variables in the first cluster (Cd and Pb) showed clearly different levels for samples on the basis of the road factor (with higher levels in milk from farms near main roads). Some differences in Cu concentrations were also observed for both classes, but with lower discriminatory capacity, while Fe and Zn showed similar or equal ranges. These results are consistent with those obtained using PCA and when the GLM was considered for testing the effect of the road factor in the metal content of milk samples.

Taking into account the high concentrations of Pb in almost 30% of the milk samples analyzed, as well as the low content of other essential mineral elements, the consumption of milk from this region may represent a health risk to the population. More extensive studies that include larger sample sizes and different metals, and that determine Pb levels in the soil, pasture, feedstuffs and water should be conducted to clarify the risk associated with consumption of milk in the study region.

## 4. Conclusions

This study’s findings indicate that raw cow’s milk from the Pernambuco State contains high levels of Pb, but low levels of the essential elements Cu, Fe, and Zn. A high percentage of samples exceeded the maximum limit for Pb established in the current Brazilian legislation. This finding was attributed to the proximity of the farms to the major roads and was confirmed by using multivariate chemometric techniques (PCA and HCA). Both unsupervised chemometric approaches demonstrated the main influence of proximity to transportation infrastructures on the metal content of milk. On the other hand, neither the impact of the presence of effluents in the vicinity of the farm or the milking method had an important effect on the metal profile of the product. Thus, according to the metal levels detected, the consumption of cow’s milk produced in this region can be considered a risk to public health due to the high levels of Pb and the low levels of essential minerals such as Cu, Zn, and Fe in some samples.

## Figures and Tables

**Table 1:** Toxic and essential trace element concentration (in mg/L) determined in milk samples in the present study.

	Mean ± SE	Median	GM	Range	25P	75P
**Cd**	0.007 ± 0.000	0.006	0.006	0.002–0.016	0.004	0.009
**Pb**	0.043 ± 0.002	0.040	0.040	0.013–0.098	0.028	0.053
**Cu**	0.020 ± 0.001	0.017	0.017	0.005–0.072	0.014	0.022
**Fe**	0.055 ± 0.004	0.041	0.040	0.002–0.283	0.026	0.073
**Zn**	0.621 ± 0.022	0.590	0.559	0.033–1.910	0.451	0.751

**Table 3:** Summary of the general linear model used to evaluate the effect of proximity of major roads (R), the presence of effluents (E), milking method (M), and their interactions on the toxic and essential trace element concentration in milk in this study (* *p* < 0.05; ** *p* < 0.01).

Element	R	E	M	R × E	R × M	E × M	R × E × M
Cd	**	−	−	−	−	−	−
Pb	**	−	−	−	−	−	−
Cu	*	*	−	−	−	−	−
Fe	−	−	−	−	−	−	−
Zn	−	−	−	−	−	−	−

**Table 4:** Eigenvalues and variance explained by the principal components obtained from PCA.

PC	Eigenvalue	% Variance Explained	% Cumulative Variance Explained
1	2.416	48.33	48.33
2	1.352	27.05	75.38
3	0.778	15.56	90.94
4	0.392	7.84	98.78
5	0.060	1.22	100.00

## Data Availability

Data sharing not applicable.
